# Superior triacylglycerol (TAG) accumulation in starchless mutants of *Scenedesmus obliquus*: (II) evaluation of TAG yield and productivity in controlled photobioreactors

**DOI:** 10.1186/1754-6834-7-70

**Published:** 2014-05-12

**Authors:** Guido Breuer, Lenny de Jaeger, Valentin P G Artus, Dirk E Martens, Jan Springer, René B Draaisma, Gerrit Eggink, René H Wijffels, Packo P Lamers

**Affiliations:** 1 Bioprocess Engineering & AlgaePARC, Wageningen University and Research Centre, P.O. Box 8129, 6700 EV Wageningen, Netherlands; 2 Food and Biobased Research & AlgaePARC, Wageningen University and Research Centre, P.O. Box 17, 6700 AA Wageningen, Netherlands; 3 Unilever Research and Development Vlaardingen, Olivier van Noortlaan 120, 3133 AT Vlaardingen, Netherlands

**Keywords:** *Scenedesmus obliquus*, *Acutodesmus obliquus*, triacylglycerol (TAG), starch, starchless, mutant

## Abstract

**Background:**

Many microalgae accumulate carbohydrates simultaneously with triacylglycerol (TAG) upon nitrogen starvation, and these products compete for photosynthetic products and metabolites from the central carbon metabolism. As shown for starchless mutants of the non-oleaginous model alga *Chlamydomonas reinhardtii*, reduced carbohydrate synthesis can enhance TAG production. However, these mutants still have a lower TAG productivity than wild-type oleaginous microalgae. Recently, several starchless mutants of the oleaginous microalga *Scenedesmus obliquus* were obtained which showed improved TAG content and productivity.

**Results:**

The most promising mutant, slm1, is compared in detail to wild-type *S. obliquus* in controlled photobioreactors. In the slm1 mutant, the maximum TAG content increased to 57 ± 0.2% of dry weight versus 45 ± 1% in the wild type. In the wild type, TAG and starch were accumulated simultaneously during initial nitrogen starvation, and starch was subsequently degraded and likely converted into TAG. The starchless mutant did not produce starch and the liberated photosynthetic capacity was directed towards TAG synthesis. This increased the maximum yield of TAG on light by 51%, from 0.144 ± 0.004 in the wild type to 0.217 ± 0.011 g TAG/mol photon in the slm1 mutant. No differences in photosynthetic efficiency between the slm1 mutant and the wild type were observed, indicating that the mutation specifically altered carbon partitioning while leaving the photosynthetic capacity unaffected.

**Conclusions:**

The yield of TAG on light can be improved by 51% by using the slm1 starchless mutant of *S. obliquus*, and a similar improvement seems realistic for the areal productivity in outdoor cultivation. The photosynthetic performance is not negatively affected in the slm1 and the main difference with the wild type is an improved carbon partitioning towards TAG.

## Background

Microalgae are well known for their ability to produce large quantities of triacylglycerol (TAG), which can be used as a resource for food, feed, and fuel production [1,2]. Microalgae-derived TAGs can be competitive to oils derived from terrestrial plants due to the higher areal productivities of microalgae and because no arable land is required for their cultivation [2]. However, the economic costs and carbon footprint of photobioreactors make it necessary to improve the areal TAG productivity even further [3,4].

The dogma on the physiological role of TAG synthesis is that TAG serves as a compact energy and carbon storage pool when formation of functional biomass is impaired. Furthermore, TAG can serve as an electron sink under unfavorable conditions for growth. An electron sink under these conditions prevents the buildup of photosynthetic products which would otherwise have resulted in over-reduction of the electron transport chains. Such over-reduction can result in the transfer of electrons to O_2_, which results in the formation of reactive oxygen species (ROSs), such as H_2_O_2_, or superoxide [5]. These ROSs can cause damage to the cell. Production of TAG can thus protect the cell against the damage induced by adverse growth conditions [6]. This is reflected by the fact that under nutrient replete conditions only trace amounts of TAG are produced. However, as a response to nitrogen starvation, TAG can be accumulated to over 40% of dry weight by many oleaginous microalgae species [6-8].

Although nitrogen starvation reduces photosynthetic efficiency [9], photosynthesis and carbon assimilation continue for a certain period when the microalgae are exposed to nitrogen depleted conditions [10]. This is supported by the observation of an up to eightfold increase in biomass dry weight concentration after nitrogen depletion in some species [7]. This increase in biomass can be explained by *de novo* production of nitrogen-free storage molecules such as TAG and carbohydrates. Even in the most oleaginous species, the increase in dry weight cannot solely be explained by the observed TAG production [7]. Other storage components such as starch are simultaneously accumulated and can easily account for over 40% of the newly produced biomass [7,11-13]. Diverting this large carbon flow away from carbohydrates towards TAG could substantially enhance TAG productivity [14].

The partitioning of assimilated carbon between TAG and carbohydrates during nitrogen starvation is a complex and highly regulated process as indicated by the activity of transcription factors and the observed changes in transcriptome and proteome during nutrient starvation [15-18]. In addition, it is often proposed that the production rates of TAG and storage carbohydrates are influenced by competition for common precursors, that is, intermediates of the central carbon metabolism such as glyceraldehyde-3-phosphate (GAP) or acetyl coenzyme A (acetyl-CoA) [19,20]. Modifying the activity of either pathway using strain improvement techniques could therefore potentially affect the carbon partitioning between TAG and storage carbohydrates [14]. One commonly attempted strategy to accomplish this is to over-express reactions in the TAG synthesis pathway, such as the initial step in fatty acid synthesis catalyzed by acetyl-CoA carboxylase [21] or the acyl transfer step catalyzed by diacylglycerol acyltransferase (DGAT) [22]. However, attempts to improve TAG production by increasing the expression of genes involved in the TAG biosynthesis pathway have been mostly unsuccessful, as reviewed by Li et al. [23].

Another commonly employed strategy is to down-regulate or inhibit the competing carbohydrate (for example starch) synthesis. Several successful attempts have been made to enhance TAG production by reducing or eliminating starch synthesis [14,23-26]. For example, Li et al. [23] observed an eightfold increase in TAG content (reaching a TAG content of 32.6% of dry weight) under mixotrophic conditions, and Li et al. [14] observed a fourfold increase in volumetric TAG productivity under photoautotrophic conditions, both using the starchless BAFJ5 mutant of *Chlamydomonas reinhardtii*.

Although cellular TAG contents are generally enhanced in starchless or impaired mutants, the overall TAG productivity of such mutant cultures is not always improved. This is because their biomass productivity under nitrogen starvation conditions is often largely reduced or even completely impaired compared to their wild-type strains [23]. This reduction in biomass productivity is often poorly characterized with the focus only directed at the TAG content, making conclusive evaluations of the mutant performance difficult. The decrease in biomass productivity could, among other possible explanations, be a result of additional mutations in, for example, the photosynthetic machinery [24] or of insufficient capacity to channel all excess photosynthate towards TAG. Especially in the latter case, the impact of starch deficiency on oleaginous microalgae might be fundamentally different from that on non-oleaginous microalgae. Namely, a starchless oleaginous microalga might be able to redirect most of the light energy that would otherwise have been used for starch synthesis towards TAG, whereas a non-oleaginous microalga might be unable to utilize this light energy. For a non-oleaginous microalga, this could thus lead to a quick buildup of photosynthetic products, which in turn could result in over-reduction of the photosynthetic machinery and the formation of harmful ROSs [5]. This might occur at a lower rate in starchless oleaginous microalgae that are able to channel most excess photosynthate towards TAG synthesis.

Most previous work on starchless mutants is performed in the non-oleaginous model alga *C. reinhardtii* and shows that the use of starchless mutants can be a feasible strategy to enhance TAG production. However, even these starchless mutants underachieve in TAG production compared to good-performing wild-type oleaginous microalgae. The TAG contents of these *C. reinhardtii* mutants are at best comparable to those of oleaginous microalgae [7,14,23]. In addition, upon nitrogen starvation their biomass productivity decreases to a much bigger extent than that of wild-type oleaginous microalgae [7,23]. Therefore, it is important to study the effect of disabling starch formation on TAG production in good-performing oleaginous microalgae.

In previous research, *Scenedesmus obliquus* (recently suggested to be reclassified to *Acutodesmus obliquus*[27]) was identified as a very promising microalga to produce TAG [7,8], and recently several starchless mutants of *S. obliquus* were obtained [28]. In shake flask studies, the slm1 mutant showed both an enhanced TAG content during initial nitrogen starvation and a biomass productivity comparable to that of the wild type, resulting in a net increase in volumetric TAG productivity [28]. In this work, a more detailed and quantitative comparison, performed in controlled photobioreactors, of the *S. obliquus* wild type and this starchless mutant is presented.

## Results and discussion

### TAG and starch accumulation

**Figure 1:**
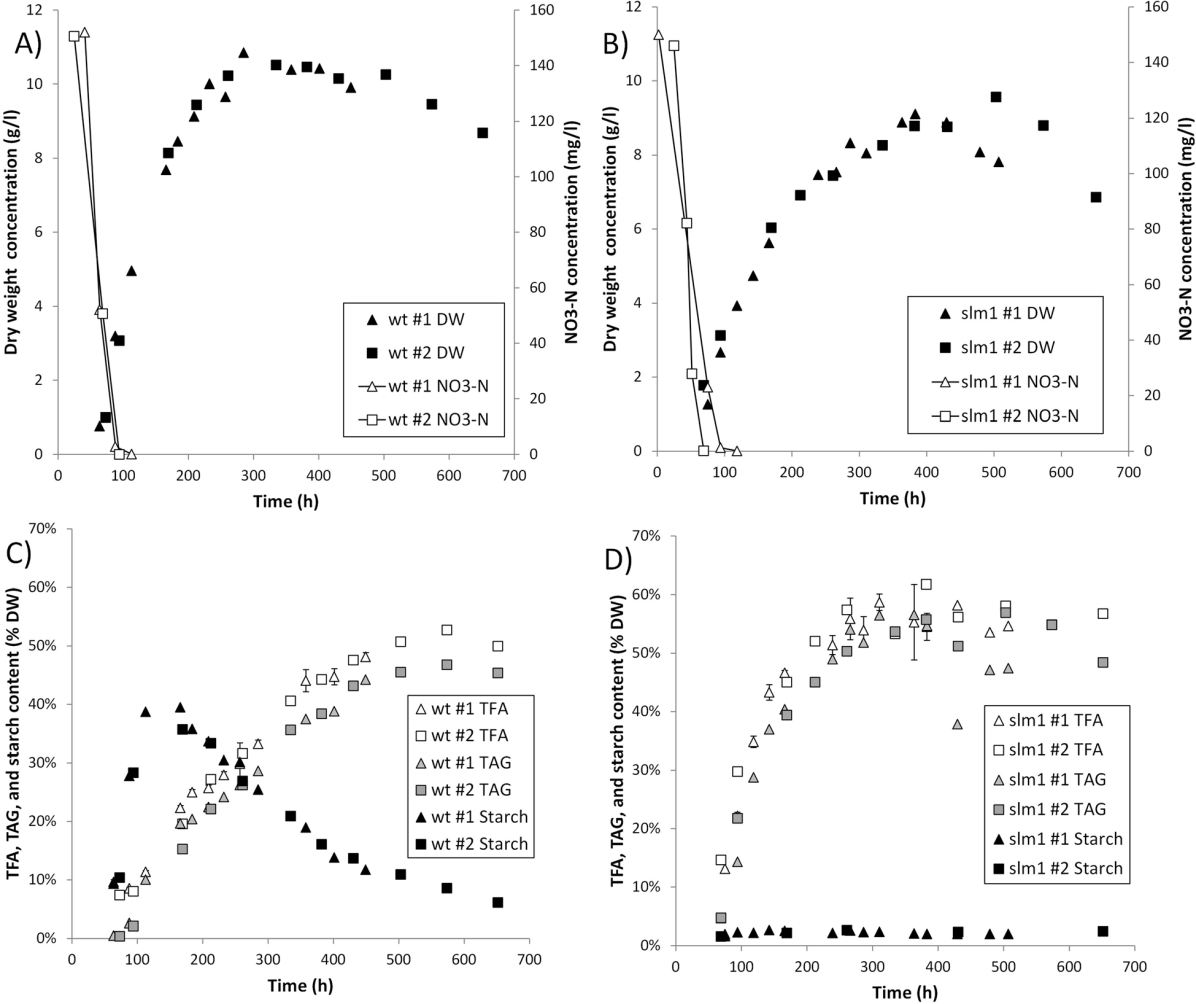
**Duplicate batch nitrogen run-out cultivations of the wt and the slm1 *S. obliquus*.** Left **(A, C)**: wild-type. Right **(B, D)**: slm1. Top **(A, B)**: biomass concentration (g DW/l) and dissolved NO_3_-N concentration. Bottom **(C, D)**: total fatty acid (TFA), TAG, and starch content. As indicated in the legend boxes in the figure, in the top figure, the black symbols represent the dry weight concentration and the open symbols represent the NO_3_-N concentration. In the bottom figures, the open symbols represent the total fatty acid content (TFA), the gray symbols represent the TAG content, and the black symbols represent the starch content. The results indicated with #1 and #2 in the figure legend represent the replicate cultivations.

For both the wild-type (wt) *S. obliquus* (UTEX 393) and a starchless mutant (slm1) of *S. obliquus*[28], duplicate nitrogen run-out experiments were performed to investigate the difference in carbon partitioning between the wt and the slm1 under nitrogen depleted conditions (Figure 1). Reactors were inoculated at 50 mg DW/l and cultivated at an incident light intensity of 100 μmol m^-2^ s^-1^ until the biomass concentration was 0.3-1 g DW/l, after which the incident light intensity was increased to 500 μmol m^-2^ s^-1^. The moment of inoculation is considered as t = 0.

Nitrogen was depleted from the culture medium at a biomass concentration of approximately 1.5-2 g/l and occurred 70 to 100 h after inoculation (Figure 1). After nitrogen was depleted, carbon assimilation and biomass formation continued, mainly as a result of accumulation of TAG (both the wt and the slm1) and starch (the wt only), which is consistent with previous observations [7,28,29]. The wt increased more rapidly in biomass concentration than the slm1 during initial nitrogen starvation and also achieved a higher maximum biomass concentration (Figure 1A,B).

**Figure 2:**
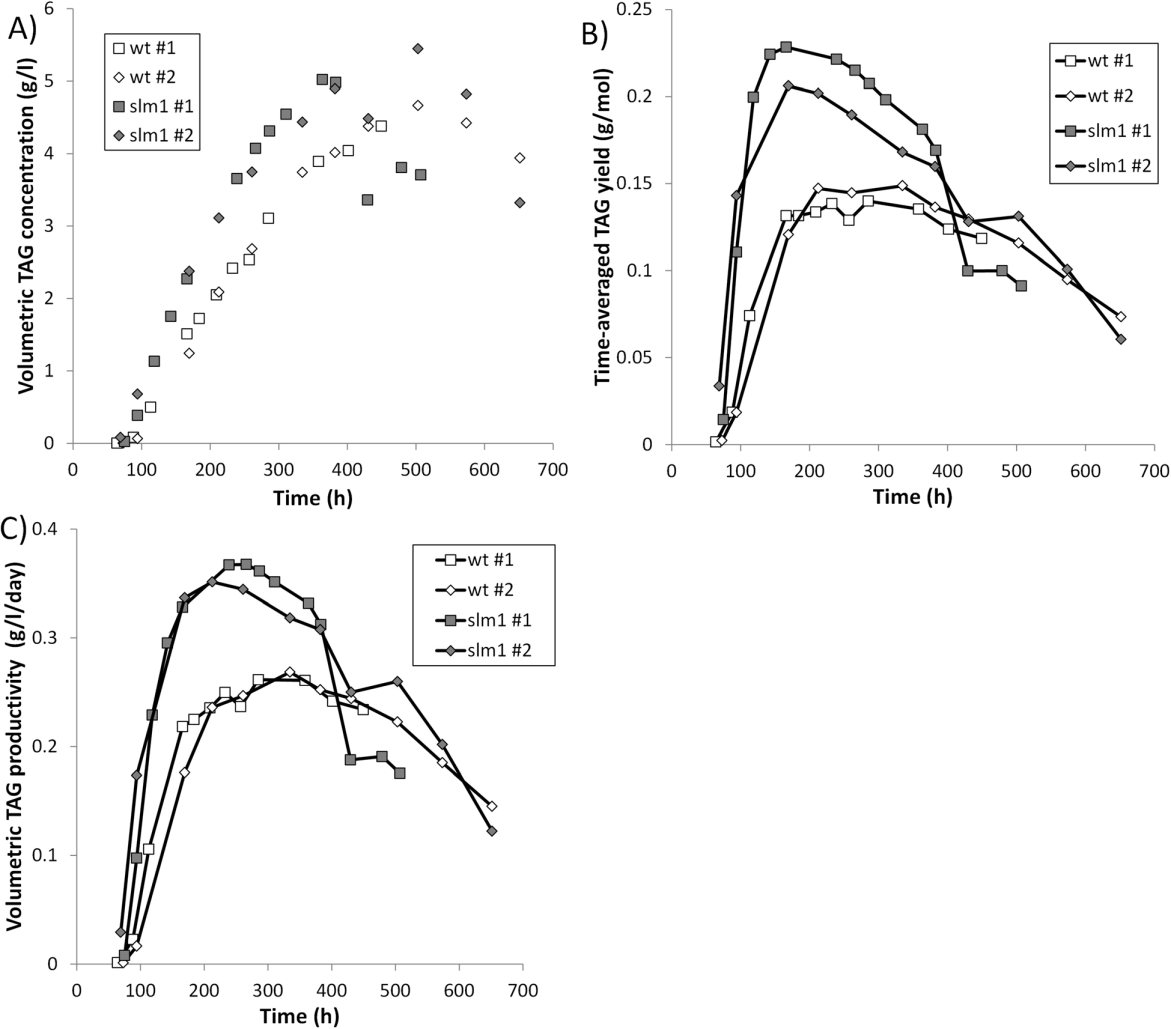
**TAG concentration (A), time-averaged yield of TAG on photons (B), and time-averaged volumetric TAG productivity (C).** Open symbols: wt. Gray symbols: slm1. The time-averaged yield and volumetric productivity are calculated over the period between inoculation and each time point. The results indicated with #1 and #2 in the figure legend represent the replicate cultivations.

In the wt cultivation, starch and TAG were accumulated simultaneously after nitrogen was depleted. Initially starch was produced at a much higher rate than TAG (Figure 1C), but when nitrogen starvation progressed starch synthesis stopped. Starch reached a maximum content of 38 ± 2% (average of duplicate cultivations ± deviation of duplicates from average) of dry weight after 168 ± 2 h and a maximum concentration of 3.6 ± 0.2 g/l after 223 ± 10 h. Subsequently starch was degraded. The starch concentration at the end of the cultivation decreased to 0.5 g/l (6% of dry weight). During this period TAG synthesis continued, and the TAG content reached a maximum of 45 ± 1% of dry weight (4.5 ± 0.1 g/l) (Figure 1C; Figure 2A). The simultaneous degradation of starch and production of TAG in the wt could indicate that degradation products of starch are used for the synthesis of TAG. This interconversion has also been suggested previously for *Pseudochlorococcum* sp. [11], *C. reinhardtii*[19], *Coccomyxa* sp. [19], and *Chlorella zofingiensis* (also known as *Chromochloris zofingiensis*) [13], as well as for conversion of chrysolaminarin into TAG in the diatom *Cyclotella cryptica*[30].

In the slm1, the production of starch is negligible. As a result, the TAG content increases more rapidly in the slm1 than in the wt during initial nitrogen starvation; the TAG content in the slm1 reached a maximum of 57 ± 0.2% of dry weight (5.2 ± 0.2 g/l) after 433 ± 70 h (Figure 1C,D; Figure 2A).

In neither the wt nor the slm1 can the combined accumulation of starch and TAG completely account for the increase in dry weight after nitrogen depletion. This difference between the measured biomass constituents and dry weight concentration is relatively constant and accounts for approximately 20 to 30% of dry weight during the entire cultivation for both the wt and the slm1. Proteins are most likely not part of this residual biomass as no nitrogen source is available for protein synthesis; also protein synthesis out of non-protein nitrogen present in the biomass can only contribute very little, because this fraction of non-protein nitrogen in the biomass is very small [31]. It is likely that the cell wall will account for a substantial part of this residual biomass. Although little is known about the cell wall composition of *S. obliquus* and other microalgae, it is hypothesized that this residual biomass consists largely of carbohydrates (other than starch) such as cellulose, which is known to be a major constituent of the cell wall of *S. obliquus* and other microalgae [32,33].

### Yields, productivity, and implications for large-scale production

Using the measured TAG concentration at each time point (Figure 2A) and the amount of light supplied specific to the reactor volume (calculated as the incident light intensity multiplied by the area-to-volume ratio of the reactor), the time-averaged yield of TAG on photons was calculated for each time point (the yield of TAG on light achieved over the period between inoculation and each time point) (Figure 2B). Because almost no TAG is produced during nitrogen replete conditions, this yield of TAG on light is very low during the initial part of the cultivation. After nitrogen depletion, the time-averaged yield increases to a maximum of 0.217 ± 0.011 and 0.144 ± 0.004 g TAG/mol photon for the slm1 and wt, respectively (Figure 2B). This illustrates that the slm1 can achieve a 51% higher time-averaged yield of TAG on light than the wt. Similarly, the maximum volumetric productivity, calculated between inoculation and each time point, was enhanced in the slm1 by 35% compared to the wt and increased from a maximum of 0.265 ± 0.004 in the wt to a maximum of 0.359 ± 0.008 g TAG l^-1^ day^-1^ in the slm1 (Figure 2C). During the period that these maxima in yield and volumetric productivity were maintained, the TAG content increased to over 40% of dry weight for both the wt and the slm1 (Figure 1C,D; Figure 2B,C).

After these maxima in yield and volumetric productivity were achieved, the difference in performance of the wt and the slm1 became smaller when the cultivation progressed. This can be explained by the degradation of starch in the wt and possible interconversion into TAG. This could enhance the TAG contents in the wt at the end of the cultivation, resulting in a smaller difference between the slm1 and wt at the end of the cultivation.

Due to the different behavior of the wt and slm1, there is a difference in the biomass concentration in the wt and slm1 cultivation (Figure 1). This did not result in a difference in light absorption rates between the wt and slm1, because nearly all light was absorbed in all cultures; therefore, a difference in the biomass concentration or pigmentation will only result in a difference in the light gradient in the photobioreactor. Furthermore, because all cultures were provided with the same amount of NO_3_, the amount of light absorbed per N-mol and per amount of catalytic biomass (assuming that the amount of catalytic biomass is proportional to the amount of nitrogen) is exactly the same.

When algae are cultivated using sunlight, the amount of light that can be provided to the photobioreactor is limited to the insolation to that area. The maximum areal productivity is therefore directly proportional to the yield on light that can be achieved. Maximizing this yield of TAG on light can therefore contribute to improving the areal productivity of microalgal TAG production. The time point where the highest time-averaged yield of TAG on light is achieved is therefore proposed as the optimum time point to harvest the culture. Previously it has been shown that this yield can be enhanced by improving the photobioreactor design [2,34] as well as optimizing cultivation conditions [29]. In this work it is shown that this yield on light can be improved by 51% by using a starchless mutant, and a similar improvement seems realistic for the areal productivity in outdoor cultivation. It should be noted that at the moment this maximum was reached, the TAG content was over 40% of the dry weight.

In this work, all cultivations were performed using continuous illumination. However, during day-night cycles starch contents in microalgae oscillate, and starch can likely provide energy for nocturnal respiration [35,36]. This might complicate cultivation of starchless mutants in day-night cycles. In higher plants such as *Arabidopsis thaliana* it is indeed reported that starchless mutants show decreased growth rates and decreased net photosynthesis rates when grown under day-night cycles, whereas these are indistinguishable from their wild types during continuous illumination [37,38]. The slm1 mutant, however, does not show decreased growth under day-night cycles under nitrogen replete conditions, and possibly the role of starch can be taken over by other storage metabolites [28]. Further investigation of the behavior of slm1 under day-night cycles and nitrogen depleted conditions would be of future interest.

### Photosynthetic energy distribution in the wt compared to the slm1

The biomass productivity was lower in the slm1 than in the wt during the initial period of nitrogen starvation. Because exactly the same amount of light was supplied, this might at first suggest a reduced photosynthetic efficiency in the slm1. However, lipids (for example, TAG) are much more energy dense than carbohydrates (37.6 kJ/g for lipids compared to 15.7 kJ/g for carbohydrates [39]). The difference in metabolic costs required to produce TAG and starch can completely explain the observed difference in biomass productivity. To illustrate this, we compare the photosynthetic requirement for the observed biomass production after nitrogen depletion in the wt and the slm1. To calculate this photosynthetic requirement, it is assumed that after nitrogen depletion only TAG, starch, and other carbohydrates (such as cell wall cellulose) are produced. The TAG and starch concentration are measured at each time point (Figure 1), and it is assumed that the remaining newly produced biomass consists of other carbohydrates (calculated as the amount of dry weight produced minus the amounts of TAG and starch produced). The photosynthetic requirement to produce the biomass that is made between nitrogen depletion and time point t can then be calculated by summing the quotients of the measured concentration of each biomass constituent at time point t and the photosynthetic yield of that biomass constituent (Eq. 1):

(1) $$\begin{array}{l} \mathrm{Photosynthetic}\;\mathrm{requirement}\left(t\right)\\ \;=\frac{C_{\mathrm{TAG}}\left(t\right)}{Y_{\mathrm{TAG},\mathrm{light}}}+\frac{C_{\mathrm{starch}}\left(t\right)}{Y_{\mathrm{starch},\mathrm{light}}}+\frac{C_{\mathrm{carbohydrate}}\left(t\right)}{Y_{\mathrm{carbohydrate},\mathrm{light}}} \end{array}$$

In Eq. 1, C_i_(t) represents the concentration of component i (g/l) at time point t and Y_i,light_ represents the photosynthetic yield of component i (g product/mol photon). These photosynthetic yields are estimated to be 1.02 g TAG/mol photon, 3.24 g starch/mol photon, and 3.24 g carbohydrate/mol photon (see Appendix A).

**Figure 3:**
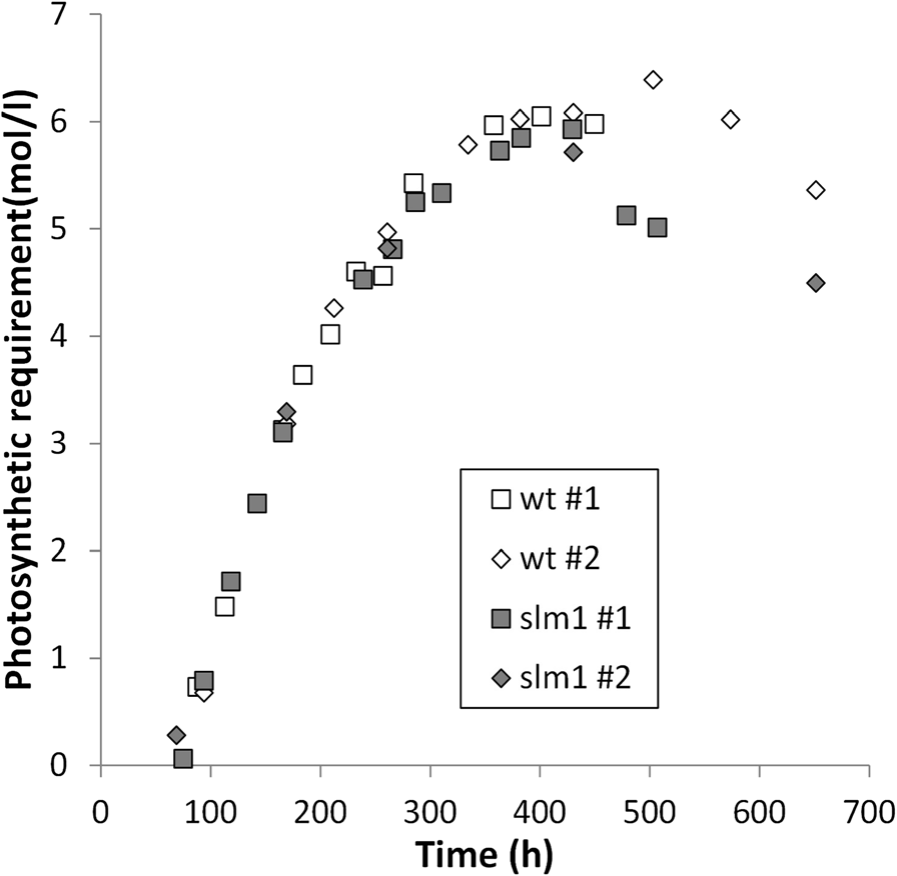
**Theoretical minimum amount of photons required to produce the observed biomass after nitrogen is depleted.** Open symbols: wt. Gray symbols: slm1. The results indicated with #1 and #2 in the figure legend represent the replicate cultivations.

Using this calculation, it appears that although the slm1 has a lower biomass productivity, the minimum photosynthetic requirement to produce that biomass is similar (Figure 3). This indicates that the slm1 does not have a reduced photosynthetic efficiency, but only seems to differ from the wt in terms of carbon partitioning.

In the wt, the calculated photosynthetic requirement also increases at the end of the cultivation, where no substantial increase in dry weight concentration is observed. This can be explained by an increase in energy density of the biomass due to a change in biomass composition (increase in TAG and decrease in starch content). This requires additional energy, which is provided by photosynthesis.

In these calculations it was assumed that the residual biomass (difference between the produced dry weight and the measured amounts of TAG and starch) consists in large part of cell wall material that is made of carbohydrates such as cellulose [33]. If this were a different biomass constituent with a different photosynthetic yield than carbohydrates, it would affect the calculated photosynthetic requirement. However, the estimated amount of this remaining fraction is similar in the wt and the slm1. Therefore, this will not result in a biased comparison.

It is observed in the wt that starch is first produced and subsequently degraded (Figure 1). This turnover is not taken into account in these calculations. However, the turnover of starch (synthesis of starch out of GAP and subsequent degradation of starch into GAP) only costs 1 ATP per glucose monomer and would only result in a minor change in the calculated photosynthetic requirement.

### Photosynthesis

**Figure 4:**
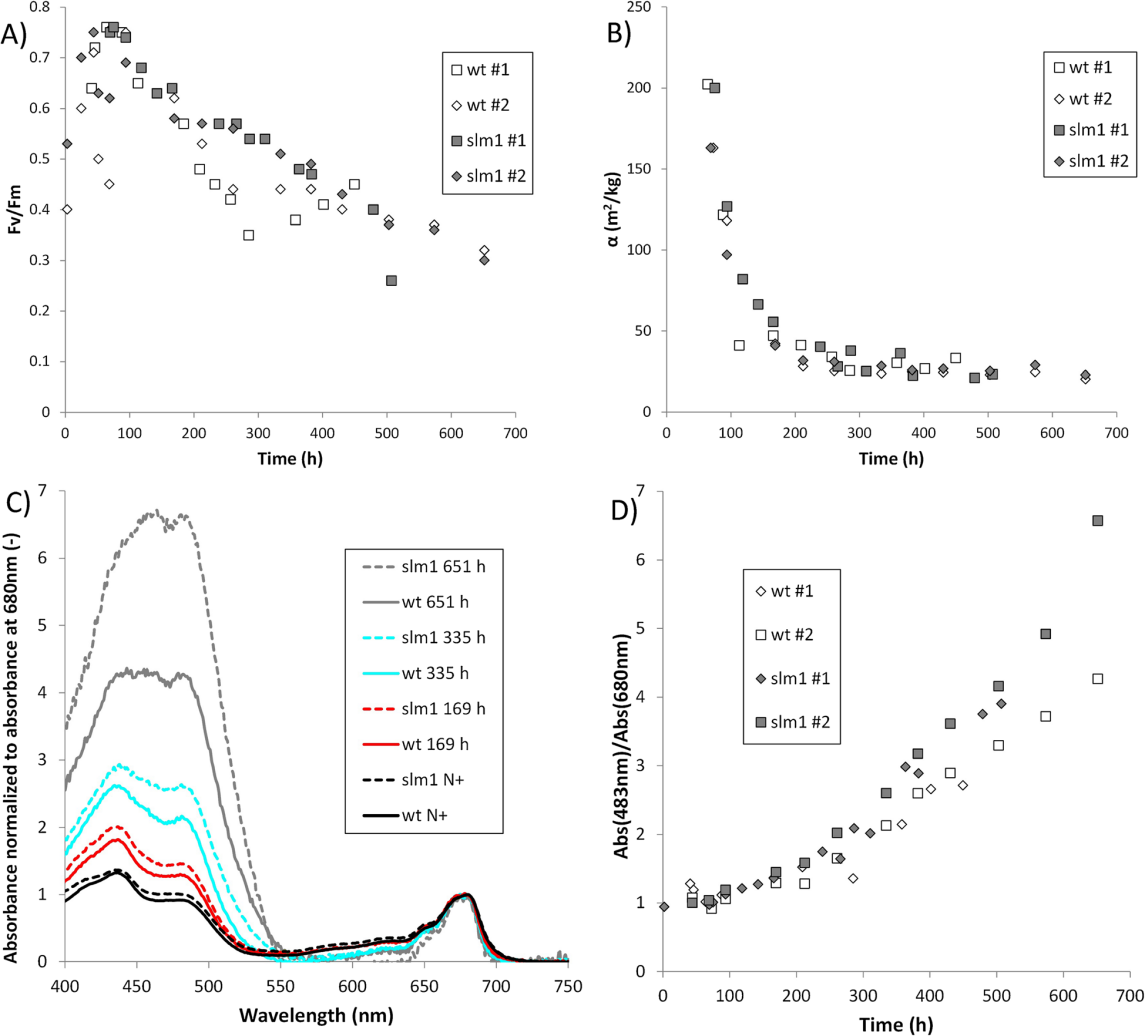
**Impact of nitrogen starvation on photosynthesis. (A)** Fv/Fm ratio. **(B)** Absorbance cross section. **(C)** Absorbance spectrum of the slm1 and wt under nitrogen replete and nitrogen depleted conditions, normalized to the absorbance at 680 nm. **(D)** Ratio of absorbance at 483 nm and 680 nm. In figure C, the time indicated in the figure legend represents the time after inoculation. Nitrogen starvation commenced 70 to 100 h after inoculation. The spectrum that is indicated with N+ in the figure legend represents the spectrum before nitrogen was depleted. Open symbols indicate the wt and gray symbols indicate the slm1. The results indicated with #1 and #2 in the figure legend represent the replicate cultivations.

The pigmentation of the cell determines the amount of light that can be absorbed and ultimately be used for photosynthesis. The absorbance cross section of the biomass was measured and used as a proxy for the pigmentation. An up to eightfold decrease in the biomass specific absorbance cross section (m^2^/g DW) was observed at the end of the cultivation compared to the point before nitrogen depletion in both the wt and the slm1 (Figure 4B). A decrease in pigmentation during nitrogen starvation is commonly observed in microalgae [40]. The volumetric absorbance cross section (m^2^/l), however, remained more or less constant throughout the entire experiment. This suggests that the decrease in biomass specific absorbance cross section is mainly a result of dilution of pigments over newly formed biomass and is likely caused to a lesser extent by net degradation of pigments.

In addition to a change in absorbance cross section, the absorbance spectrum, and thus the pigment class composition, changed drastically (Figure 4C). Photoprotective pigments (carotenoids) can be produced in response to physiological stress to prevent photo-oxidative damage [40,41]. The ratio of chlorophyll over carotenoids decreased during nitrogen starvation as is apparent from the increase in absorbance at 483 nm (the observed absorbance maximum of carotenoids) relative to the absorbance at 680 nm (the observed absorbance maximum of chlorophyll) (Figure 4D). The decrease in absorbance cross section between the slm1 and wt was similar, but the slm1 showed a higher ratio of absorbance at 483 nm/680 nm as the nitrogen starvation progressed. This suggests that the slm1 has relatively more carotenoids than the wt. This difference between the slm1 and wt became more apparent when nitrogen starvation progressed (Figure 4D). These observations suggest that a progressively smaller fraction of the absorbed light is available for photosynthesis when nitrogen starvation progresses due to an increased carotenoid/chlorophyll ratio.

The variable fluorescence/maximum fluorescence ratio (Fv/Fm) was measured and can be used as a proxy for the intrinsic (or maximum) PSII quantum yield [10,23]. Although it does not directly reflect the photosynthetic efficiency achieved in the photobioreactor, it is often used as a diagnostic value for the photosynthetic performance [42]. Immediately after inoculation, the Fv/Fm ratio started substantially below the maximum value that was observed (Figure 4A). This could possibly be due to a shock in biomass concentration and light intensity as a result of inoculation. During the nitrogen replete growth phase, the Fv/Fm ratio increased gradually to a maximum of 0.78, which is consistent with maximum values observed in other studies [23]. Once nitrogen was depleted, the Fv/Fm ratio gradually decreased, as is commonly observed [10,23]. This could be an indication of increased damage to the photosystems. Fv/Fm ratios in the slm1 are comparable to or even slightly higher than the Fv/Fm ratios in the wt. This is consistent with the observation presented in Figure 3, that the photosynthetic performance is not negatively affected in the slm1 and that the main difference with the wt is an improved carbon partitioning towards TAG in the slm1.

## Conclusions

The maximum TAG content increased from 45 ± 1% in wild-type to 57 ± 0.2% of dry weight in starchless *S. obliquus* (slm1). The slm1 had a lower biomass productivity, which can completely be explained by the higher energy requirement to produce TAG compared to starch. The maximum yield of TAG on light in the mutant increased from 0.144 ± 0.004 to 0.217 ± 0.011 g TAG/mol photon, and a 51% improvement in areal TAG productivity therefore seems realistic for outdoor cultivation. This work highlights the potential of improved carbon partitioning using a starchless mutant to increase TAG productivity in oleaginous microalgae.

## Methods

### Strains, pre-culture conditions, and cultivation medium

Wild-type (wt) *S. obliquus* UTEX 393 (recently suggested to be reclassified to *Acutodesmus obliquus*[27]) was obtained from the University of Texas Culture collection of algae (UTEX). The starchless mutant (slm1) was obtained using UV radiation-induced random mutagenesis on the wild-type strain of *S. obliquus*[28]. The culture medium was similar to that described by Breuer et al. [29] with the exception that all vitamins were omitted from the culture medium. The culture medium was autotrophic and contained 10 mM KNO_3_ as the limiting nutrient. All other required nutrients were present in excess. Pre-cultures were maintained in 16:8 h light:dark cycles as described by Breuer et al. [7]. Both the wt and the slm1 were able to continue growing after multiple serial dilutions while being cultivated autotrophically under these day-night cycles (16:8 light:dark).

### Experimental conditions

Batch cultivations were performed in flat-panel airlift-loop photobioreactors with a working volume of 1.7 l (Labfors 5 Lux, Infors HT, Switzerland). The reactor design is similar to that described by Klok et al. [9]. The reactors were sparged with air containing 2% CO_2_ at 1 l/min. The reactors were continuously illuminated (24 h/day) using LED lamps with a warm white spectrum located on the culture side of the reactor. The incident light intensity was calibrated by measuring the average light intensity on the culture side of the front glass plate. The light path (reactor depth) was 2 cm. The temperature was controlled at 27.5°C and the pH was controlled at pH 7 using automatic addition of 1 M HCl. These values for pH and temperature were found to be optimal for both growth and TAG accumulation in *S. obliquus* in previous research [29]. A few milliliters of a 1% Antifoam B solution (J. T. Baker) were added manually when excessive foaming was visible. Prior to inoculation, reactors were heat-sterilized and subsequently filled with 0.2 μm filter-sterilized medium. Reactors were inoculated at 50 mg algae dry matter/l and grown at an incident light intensity of 100 μmol m^-2^ s^-1^ until the biomass density reached 0.3 to 1 g dry weight (DW)/l (typically after 48 h). At this point the incident light intensity was increased to 500 μmol m^-2^ s^-1^. Periodically, samples were taken aseptically and analyzed for dry weight concentration, biomass composition, residual dissolved nitrate, Fv/Fm ratio, absorbance spectrum, and absorbance cross section. After nitrogen was depleted, more than 95% of the incident light was absorbed. Therefore, for simplicity, in the calculations it is assumed that the absorbed light is identical to the incident light intensity.

Although a condenser was installed at the gas exhaust of the reactor, water losses were present due to evaporation. Evaporation was quantified in a separate reactor that was filled with water and operated under the same conditions as during cultivation. The evaporation rate was 0.9 ml/h. Evaporation results in concentration of the biomass, which thus has an effect on the measured dry weight concentration. The measured biomass composition is unaffected. The biomass concentration was corrected for evaporation using $C_{x,\mathrm{corrected}}=C_{x,\mathrm{oberved}}\frac{V_{\mathrm{observed}}}{V_{\mathrm{without}\;\mathrm{evaporation}}}$. The correction factor decreased from 1 at the start of the cultivation to a minimum between 0.60 and 0.71 (depending on the duration of the cultivation) at the end of the cultivation. All presented results and calculations throughout this work are based on the concentrations corrected for evaporation. Evaporation rates were similar in all experiments and therefore did not result in a biased comparison between the slm1 and wt in any way. However, evaporation and the accompanying concentration effect did increase the steepness of the light gradient in the reactor and reduced light penetration and biomass specific light absorption rates.

### Analyses

#### Dry weight

The dry weight concentration was determined by filtrating culture broth over preweighted glass fiber filters and measuring the weight increase of the filters after drying at 95°C as described by Kliphuis et al. [43].

#### Total fatty acid

The total fatty acid (TFA) concentration was determined by a sequence of cell disruption, total lipid extraction in chloroform:methanol, transesterification of acyl lipids to fatty acid methyl esters (FAMEs), and quantification of FAMEs using gas chromatography as described by Breuer et al. [44]. Tripentadecanoin was used as an internal standard.

#### TAG

The TAG concentration was determined by separating the total lipid extract, obtained using the aforementioned method, into a TAG and polar lipid pool using a solid phase extraction column (SPE) as described by Breuer et al. [29], followed by transesterification and quantification of the fatty acids in the TAG pool as described by Breuer et al. [44].

#### Starch

The starch concentration was determined using an AA/AMG Total Starch Kit (Megazyme, Ireland) with modifications as described by de Jaeger et al. [28]. The procedure consisted of a sequence of cell disruption, starch precipitation using an aqueous solution of 80% ethanol, enzymatic hydrolysis of starch to glucose monomers using α-amylase and amyloglucosidase, and a spectrophotometric-based assay for quantification of glucose monomers.

#### Dissolved nitrate

Dissolved nitrate was analyzed in supernatant using a Seal analytical AQ2 nutrient analyzer (SEAL Analytical Inc., USA) according to the manufacturer’s instructions.

#### Fv/Fm

Pulse amplitude modulation (PAM) fluorometry was used to determine the Fv/Fm ratio using an AquaPen AP-100 fluorescence spectrophotometer (PSI, Czech Republic) according to the manufacturer’s instructions. Cultures were diluted in demineralized water to an optical density of 0.4 at 750 nm in a cuvette with a light path of 10 mm (equivalent to a concentration of 0.2 g DW/l) and adapted to dark conditions for 15 min prior to the measurement. It was confirmed that longer dark-adaptation times did not affect the results.

#### Absorbance spectrum and absorbance cross section

Cell suspensions were diluted to an optical density at 750 nm of 1.4 to1.6 as measured in a cuvette with a 1 cm light path. Subsequently, the absorbance spectrum was measured in these diluted cell suspensions with a Shimadzu UV-2600 integrating sphere spectrophotometer in the spectrum 300 to 750 nm, which results in the absorbance spectrum corrected for scattering. Residual scattering was calculated as the average absorbance between 740 and 750 nm and subtracted from the absorbance spectrum. From this absorbance spectrum, the average biomass dry weight specific absorbance cross section between 400 and 700 nm (α, unit: m^2^/g) was calculated as $\alpha =\frac{\sum_{400}^{700}\mathrm{ab}s_{\lambda }\frac{\ln\left(10\right)}{z}}{300\;\mathrm{DW}}$ , where abs_λ_ is the absorbance at wavelength λ, z the light path of the cuvette (0.002 m), and DW the dry weight concentration (g/m^3^).

### Appendix A Calculation of the photosynthetic yield of biomass constituents

TAG consists of a glycerol backbone with three fatty acids. Because approximately 50% of the fatty acids in TAG are oleic acid molecules in *S. obliquus*[29], triolein is used as the reference TAG molecule to calculate the photosynthetic yield of TAG. In the production of one molecule of oleic acid from CO_2_, 70 molecules of nicotinamide adenine dinucleotide phosphate (NADPH) and 71 molecules of ATP are required and 18 molecules of nicotinamide adenine dinucleotide (NADH) are produced. In the production of the glycerol backbone 7 NADPH and 9 ATP are consumed [45]. Finally, in the condensation of the fatty acids to the glycerol backbone, it is assumed that 1 ATP is consumed for each fatty acid. This results in the net utilization of cofactors as presented in Eq. A1:

(A1) $$\begin{array}{l} 217\;\mathrm{NADPH}+225\;\mathrm{ATP}\\ \;+54\;\mathrm{NA}D^{+}\to 1\;\mathrm{triolein}+217\;\mathrm{NAD}P^{+}\\ \;+54\;\mathrm{NADH}+225\;\mathrm{ADP}+225P_{i} \end{array}$$

Similarly, one glucose monomer of starch can be produced using 19 ATP and 12 NADPH (Eq. A2). It is assumed that production of other carbohydrates (such as cell wall cellulose) is similar to production of starch in terms of metabolic requirements.

(A2) $$\begin{array}{l} 12\;\mathrm{NADPH}\\ \;+19\;\mathrm{ATP}\to 1\;\mathrm{starch}\;\mathrm{glucose}\;\mathrm{monomer}\\ \;+12\;{\mathrm{NADP}}^{+}+19\;\mathrm{ADP}+19\;P_{i} \end{array}$$

The required cofactors can be provided using photosynthesis through either linear electron transport (theoretical maximum: 8 photons → 3 ATP + 2 NADPH) or cyclic electron transport (theoretical maximum: 2 photons → 1 ATP). Furthermore, it is assumed that oxidative phosphorylation can be used to provide 2.5 ATP using 1 NADH.

Using these stoichiometric relationships, it can be calculated that a minimum of 868 mol photons are required to produce 1 mol of triolein and a minimum of 50 mol photons are required to produce 1 mol of starch glucose monomers. Using the molecular weights of triolein (885 g/mol) and starch glucose monomers (162 g/mol), theoretical maximum yields of 1.02 g TAG/mol photon and 3.24 g starch/mol photon can be found. Note that according to the stoichiometric relationship of triolein production, additional NADH is produced (Eq. A1). If this NADH can be used to reduce NADP^+^ to NADPH, the yield of TAG on photons could increase to 1.36 g TAG/mol photon.

## Abbreviations

DW: dry weight; TAG: triacylglycerol; wt: wild-type; ROS: reactive oxygen species; FAME: fatty acid methyl ester; TFA: total fatty acids; PAM: pulse amplitude modulation; SPE: solid phase extraction.

## Competing interests

RD is employed by Unilever; this does not alter the authors’ adherence to the Biotechnology for Biofuels policies on sharing data and materials. This study has been carried out in R&D collaboration between Wageningen UR and Unilever R&D Vlaardingen. In general, Unilever is interested in the potential of microalgae as an alternative sustainable source of oils. All other authors declare that they have no competing interests.

## Authors’ contributions

LJ, JS, and GE generated and selected the mutant. GB, LJ, VA, PL, and DM designed the experiments. VA and GB performed the experiments. VA, GB, LJ, PL, and DM interpreted the data. GB and LJ wrote the manuscript. PL, DM, RD, JS, GE, and RW supervised and edited the manuscript. All authors read and approved the final manuscript.

## References

[B1] Draaisma RB Wijffels RH Slegers PM Brentner LB Roy A Barbosa MJ Food commodities from microalgae Curr Opin Biotechnol 2013 24 169 177 23084075 10.1016/j.copbio.2012.09.012

[B2] Wijffels RH Barbosa MJ An outlook on microalgal biofuels Science 2010 329 796 799 20705853 10.1126/science.1189003

[B3] Norsker N-H Barbosa MJ Vermuë MH Wijffels RH Microalgal production — a close look at the economics Biotechnol Adv 2011 29 24 27 20728528 10.1016/j.biotechadv.2010.08.005

[B4] Brentner LB Eckelman MJ Zimmerman JB Combinatorial life cycle assessment to inform process design of industrial production of algal biodiesel Environ Sci Technol 2011 45 7060 7067 21662987 10.1021/es2006995

[B5] Asada K Production and scavenging of reactive oxygen species in chloroplasts and their functions Plant Physiol 2006 141 391 396 16760493 10.1104/pp.106.082040 PMC1475469

[B6] Hu Q Sommerfeld M Jarvis E Ghirardi M Posewitz M Seibert M Darzins A Microalgal triacylglycerols as feedstocks for biofuel production: perspectives and advances Plant J 2008 54 621 639 18476868 10.1111/j.1365-313X.2008.03492.x

[B7] Breuer G Lamers PP Martens DE Draaisma RB Wijffels RH The impact of nitrogen starvation on the dynamics of triacylglycerol accumulation in nine microalgae strains Bioresour Technol 2012 124 217 226 22995162 10.1016/j.biortech.2012.08.003

[B8] Griffiths MJ Hille RP Harrison STL Lipid productivity, settling potential and fatty acid profile of 11 microalgal species grown under nitrogen replete and limited conditions J Appl Phycology 2011 24 989 1001

[B9] Klok AJ Martens DE Wijffels RH Lamers PP Simultaneous growth and neutral lipid accumulation in microalgae Bioresour Technol 2013 134 233 243 23500580 10.1016/j.biortech.2013.02.006

[B10] Simionato D Block MA La Rocca N Jouhet J Maréchal E Finazzi G Morosinotto T The response of Nannochloropsis gaditana to nitrogen starvation includes de novo biosynthesis of triacylglycerols, a decrease of chloroplast galactolipids, and reorganization of the photosynthetic apparatus Eukaryot Cell 2013 12 665 676 23457191 10.1128/EC.00363-12 PMC3647774

[B11] Li Y Han D Sommerfeld M Hu Q Photosynthetic carbon partitioning and lipid production in the oleaginous microalga Pseudochlorococcum sp. (Chlorophyceae) under nitrogen-limited conditions Bioresour Technol 2011 102 123 129 20594832 10.1016/j.biortech.2010.06.036

[B12] Klok AJ Verbaanderd JA Lamers PP Martens DE Rinzema A Wijffels RH A model for customising biomass composition in continuous microalgae production Bioresour Technol 2013 146 89 100 23911819 10.1016/j.biortech.2013.07.039

[B13] Zhu S Huang W Xu J Wang Z Xu J Yuan Z Metabolic changes of starch and lipid triggered by nitrogen starvation in the microalga Chlorella zofingiensis Bioresour Technol 2013 152 292 298 24308944 10.1016/j.biortech.2013.10.092

[B14] Li Y Han D Hu G Dauvillee D Sommerfeld M Ball S Hu Q Chlamydomonas starchless mutant defective in ADP-glucose pyrophosphorylase hyper-accumulates triacylglycerol Metab Eng 2010 12 387 391 20172043 10.1016/j.ymben.2010.02.002

[B15] Boyle NR Page MD Liu B Blaby IK Casero D Kropat J Cokus SJ Hong-Hermesdorf A Shaw J Karpowicz SJ Gallaher SD Johnson S Benning C Pellegrini M Grossman A Merchant SS Three acyltransferases and nitrogen-responsive regulator are implicated in nitrogen starvation-induced triacylglycerol accumulation in Chlamydomonas J Biol Chem 2012 287 15811 15825 22403401 10.1074/jbc.M111.334052 PMC3346115

[B16] Dong H-P Williams E Wang D-z Xie Z-X Hsia R-c Jenck A Halden R Li J Chen F Place AR Responses of Nannochloropsis oceanica IMET1 to long-term nitrogen starvation and recovery Plant Physiol 2013 162 1110 1126 23637339 10.1104/pp.113.214320 PMC3668043

[B17] Valenzuela J Mazurie A Carlson R Gerlach R Cooksey K Peyton B Fields M Potential role of multiple carbon fixation pathways during lipid accumulation in Phaeodactylum tricornutum Biotechnol for Biofuels 2012 5 1 17 10.1186/1754-6834-5-40 PMC3457861 22672912

[B18] Blaby IK Glaesener AG Mettler T Fitz-Gibbon ST Gallaher SD Liu B Boyle NR Kropat J Stitt M Johnson S Benning C Pellegrini M Casero D Merchant SS Systems-level analysis of nitrogen starvation-induced modifications of carbon metabolism in a Chlamydomonas reinhardtii starchless mutant The Plant Cell 2013 Online 10.1105/tpc.113.117580 PMC3875720 24280389

[B19] Msanne J Xu D Konda AR Casas-Mollano JA Awada T Cahoon EB Cerutti H Metabolic and gene expression changes triggered by nitrogen deprivation in the photoautotrophically grown microalgae Chlamydomonas reinhardtii and Coccomyxa sp. C-169 Phytochemistry 2012 75 50 59 22226037 10.1016/j.phytochem.2011.12.007

[B20] Fan J Yan C Andre C Shanklin J Schwender J Xu C Oil accumulation is controlled by carbon precursor supply for fatty acid synthesis in Chlamydomonas reinhardtii Plant Cell Physiol 2012 53 1380 1390 22642988 10.1093/pcp/pcs082

[B21] Dunahay TG Jarvis EE Dais SS Roessler PG Manipulation of microalgal lipid production using genetic engineering Applied Biochemistry and Biotechnology 1996 57–58 223 231

[B22] La Russa M Bogen C Uhmeyer A Doebbe A Filippone E Kruse O Mussgnug JH Functional analysis of three type-2 DGAT homologue genes for triacylglycerol production in the green microalga Chlamydomonas reinhardtii J Biotechnol 2012 162 13 20 22542934 10.1016/j.jbiotec.2012.04.006

[B23] Li Y Han D Hu G Sommerfeld M Hu Q Inhibition of starch synthesis results in overproduction of lipids in Chlamydomonas reinhardtii Biotechnol Bioeng 2010 107 258 268 20506159 10.1002/bit.22807

[B24] Siaut M Cuiné S Cagnon C Fessler B Nguyen M Carrier P Beyly A Beisson F Triantaphylidès C Li-Beisson Y Peltier G Oil accumulation in the model green alga Chlamydomonas reinhardtii: characterization, variability between common laboratory strains and relationship with starch reserves BMC Biotechnol 2011 11 7 21255402 10.1186/1472-6750-11-7 PMC3036615

[B25] Wang ZT Ullrich N Joo S Waffenschmidt S Goodenough U Algal lipid bodies: stress induction, purification, and biochemical characterization in wild-type and starchless Chlamydomonas reinhardtii Eukaryot Cell 2009 8 1856 1868 19880756 10.1128/EC.00272-09 PMC2794211

[B26] Ramazanov A Ramazanov Z Isolation and characterization of a starchless mutant of Chlorella pyrenoidosa STL-PI with a high growth rate, and high protein and polyunsaturated fatty acid content Phycological Res 2006 54 255 259

[B27] Krienitz L Bock C Present state of the systematics of planktonic coccoid green algae of inland waters Hydrobiologia 2012 698 295 326

[B28] de Jaeger L Verbeek R Springer J Eggink G Wijffels RH Superior triacylglycerol (TAG) accumulation in starchless mutants of Scenedesmus obliquus: (I) mutant generation and characterisation Biotechnology for Biofuels 2014 7 69 24920957 10.1186/1754-6834-7-69 PMC4052810

[B29] Breuer G Lamers PP Martens DE Draaisma RB Wijffels RH Effect of light intensity, pH, and temperature on triacylglycerol (TAG) accumulation induced by nitrogen starvation in Scenedesmus obliquus Bioresour Technol 2013 143 1 9 23774290 10.1016/j.biortech.2013.05.105

[B30] Roessler PG Effects of silicon deficiency on lipid-composition and metabolism in the diatom Cyclotella cryptica J Phycol 1988 24 394 400

[B31] Becker EW Micro-algae as a source of protein Biotechnol Adv 2007 25 207 210 17196357 10.1016/j.biotechadv.2006.11.002

[B32] Aguirre A-M Bassi A Investigation of biomass concentration, lipid production, and cellulose content in Chlorella vulgaris cultures using response surface methodology Biotechnol Bioeng 2013 110 2114 2122 23436332 10.1002/bit.24871

[B33] Burczyk J Grzybek H Banaś J Banaś E Presence of cellulase in the algae Scenedesmus Exp Cell Res 1970 63 451 453 5490343 10.1016/0014-4827(70)90236-3

[B34] Cuaresma M Janssen M Vílchez C Wijffels RH Horizontal or vertical photobioreactors? how to improve microalgae photosynthetic efficiency Bioresour Technol 2011 102 5129 5137 21334888 10.1016/j.biortech.2011.01.078

[B35] de Winter L Klok AJ Cuaresma Franco M Barbosa MJ Wijffels RH The synchronized cell cycle of Neochloris oleoabundans and its influence on biomass composition under constant light conditions Algal Research 2013 2 4 313 320

[B36] Ral J-P Colleoni C Wattebled F Dauvillée D Nempont C Deschamps P Li Z Morell MK Chibbar R Purton S d’Hulst C Bass SG Circadian clock regulation of starch metabolism establishes GBSSI as a major contributor to amylopectin synthesis in Chlamydomonas reinhardtii Plant Physiol 2006 142 305 317 16844835 10.1104/pp.106.081885 PMC1557617

[B37] Caspar T Huber SC Somerville C Alterations in growth, photosynthesis, and respiration in a starchless mutant of Arabidopsis thaliana (l.) deficient in chloroplast phosphoglucomutase activity Plant Physiol 1985 79 11 17 16664354 10.1104/pp.79.1.11 PMC1074821

[B38] Radakovits R Jinkerson RE Darzins A Posewitz MC Genetic engineering of algae for enhanced biofuel production Eukaryot Cell 2010 9 486 501 20139239 10.1128/EC.00364-09 PMC2863401

[B39] Jakob T Wagner H Stehfest K Wilhelm C A complete energy balance from photons to new biomass reveals a light- and nutrient-dependent variability in the metabolic costs of carbon assimilation J Exp Bot 2007 58 2101 2112 17483116 10.1093/jxb/erm084

[B40] Geider R Macintyre H Graziano L McKay RM Responses of the photosynthetic apparatus of Dunaliella tertiolecta (Chlorophyceae) to nitrogen and phosphorus limitation Eur J Phycology 1998 33 315 332

[B41] Lamers PP Janssen M De Vos RCH Bino RJ Wijffels RH Exploring and exploiting carotenoid accumulation in Dunaliella salina for cell-factory applications Trends Biotechnol 2008 26 631 638 18752860 10.1016/j.tibtech.2008.07.002

[B42] Maxwell K Johnson GN Chlorophyll fluorescence—a practical guide J Exp Bot 2000 51 659 668 10938857 10.1093/jxb/51.345.659

[B43] Kliphuis AMJ Klok AJ Martens DE Lamers PP Janssen M Wijffels RH Metabolic modeling of Chlamydomonas reinhardtii: energy requirements for photoautotrophic growth and maintenance J Appl Phycology 2011 24 253 266 10.1007/s10811-011-9674-3 PMC3289792 22427720

[B44] Breuer G Evers WAC de Vree JH Kleinegris DMM Martens DE Wijffels RH Lamers PP Analysis of fatty acid content and composition in microalgae J Vis Exp 2013 80 e50628 10.3791/50628 PMC3938209 24121679

[B45] Johnson X Alric J Central carbon metabolism and electron transport in Chlamydomonas reinhardtii, metabolic constraints for carbon partitioning between oil and starch Eukaryot Cell 2013 12 776 793 23543671 10.1128/EC.00318-12 PMC3675994

